# Safety of Robot‐Assisted Radical Cystectomy for Muscle‐Invasive Bladder Cancer: A Real‐World Analysis Using the National Clinical Database in Japan

**DOI:** 10.1002/cam4.71109

**Published:** 2025-08-02

**Authors:** Shingo Hatakeyama, Hiroyuki Yamamoto, Hiroshi Kitamura, Toshiyuki Kamoto, Eiji Kikuchi

**Affiliations:** ^1^ National Clinical Database Steering Committee of Japanese Urological Association Tokyo Japan; ^2^ Department of Urology Hirosaki University Graduate School of Medicine Hirosaki Japan; ^3^ Department of Healthcare Quality Assessment, Graduate School of Medicine The University of Tokyo Tokyo Japan; ^4^ Faculty of Medicine, Department of Urology University of Toyama Toyama Japan; ^5^ Faculty of Medicine, Department of Urology University of Miyazaki Miyazaki Japan; ^6^ Department of Urology St. Marianna University School of Medicine Kawasaki Kanagawa Japan

**Keywords:** bladder cancer, laparoscopic radical cystectomy, radical prostatectomy, robot‐assisted, surgery

## Abstract

**Background:**

Radical cystectomy (RC) is a standard‐of‐care treatment for muscle‐invasive bladder cancer (MIBC). However, there is a lack of data comparing the benefit of robot‐assisted radical cystectomy (RARC) with laparoscopic radical cystectomy (LRC) or open radical cystectomy (ORC) in Japan. We aimed to explore the safety of RARC compared with that of LRC or ORC using the Japanese National Clinical Database (NCD).

**Methods:**

This retrospective study reviewed the data of 17,549 patients with MIBC who underwent radical cystectomy between February 2019 and December 2022 in the Japanese NCD. We compared the postoperative complications within 30 days between the surgical procedures using the Clavien–Dindo classification. Multivariable logistic regression analysis was conducted for grade ≥ 3 postoperative complications.

**Results:**

Overall, 8308, 3101, and 6140 patients underwent RARC, LRC, and ORC, respectively. The rates of any grade and grade ≥ 3 postoperative complications within 30 days were significantly lower in the RARC group than in the ORC group. RARC had no significant difference in grade ≥ 3 postoperative complications in the ileal neobladder. Regarding any grade postoperative complications, RARC had a significantly lower rate of delirium, surgical site infection, sepsis, and gastrointestinal tract leakage than ORC. Multivariable logistic regression analysis revealed that the incidence of grade ≥ 3 postoperative complications was significantly lower in RARC (odds ratio, 0.83; 95% confidence interval, 0.72–0.95).

**Conclusions:**

RARC had a lower complication rate compared with ORC. LRC had comparable outcomes to RARC. The findings of this study contribute to RARC as being the standard treatment for MIBC.

## Graphical abstract

We assessed the clinical benefit of robot‐assisted radical cystectomy (RARC) compared to other procedures in real‐world practice. The advantages of RARC over open radical cystectomy were lower postoperative complication rates in real‐world practice.

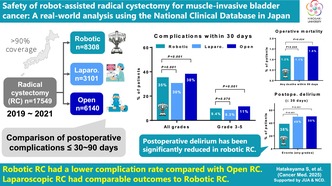

## 1 Introduction

Radical cystectomy (RC) is the standard therapy for muscle‐invasive bladder cancer (MIBC) [1, 2]. According to several guidelines, the skill of the surgeon and the volume of procedures performed at the facility are important factors, regardless of whether the procedure is robotic or open [1, 2]. However, its mortality rate is high and comparable to that for total esophagectomy [3]. Despite multidisciplinary treatment such as neoadjuvant therapy, extended lymph node dissection, and adjuvant therapy, the 5‐year survival rate following RC is only 50%–60% [4, 5]. Robot‐assisted RC (RARC) is a minimally invasive procedure that may prevent complications and improve prognosis [6, 7, 8, 9, 10]. A Phase III randomized trial (RAZOR trial) revealed that RARC was superior to open RC (ORC) in terms of blood loss, blood transfusion rate, and length of hospital stay, but the prognosis and rate of complications were comparable between the two procedures [11, 12]. Thus, it is necessary to verify whether the same results can be obtained in real‐world practice, wherein patients are ineligible for trial participation. Moreover, we cannot directly translate the outcomes of clinical trials into clinical practice in Japan due to the difference in the medical insurance system and medical risk related to different races [13, 14, 15, 16]. Therefore, large‐scale real‐world data are required to explore the real‐world benefits of RARC in Japan.

The Japanese National Clinical Database (NCD) is a large‐scale, nationwide, web‐based data entry system established in 2010 that is linked to the surgical board certification system and covers most surgeries (90%–95%) performed in Japan [17]. The NCD registration in urology was launched in April 2018, at the time of the insurance coverage of RARC for MIBC [18, 19]. All RC cases registered detailed data via a specific platform. Thus, the NCD includes most of the real‐world RC cases in Japan. Herein, we report the safety of RARC for MIBC in Japan compared to laparoscopic RC (LRC) and open RC (ORC) using the NCD.

## 2 Methods

### 2.1 Study Design and Ethics Statement

This retrospective nationwide study was performed according to the ethical standards of the Declaration of Helsinki. This study was approved by the Ethics and Conflict of Interest Committee of the National Clinical Database and the Hirosaki University School of Medicine (Authorization No. 2022‐084‐1). According to the provisions of the ethics committee and the ethics guidelines in Japan, written informed consent is not required for retrospective and/or observational studies using materials such as existing documents, and only public disclosure of study information is needed (opt‐out approach).

### 2.2 Patient Selection and Evaluation

Using the NCD, we reviewed RC cases performed between February 2019 and December 2022 from 1185 facilities nationwide under the initiative of the JUA. We evaluated the characteristics of patients from a case report form and included age, sex, Eastern Cooperative Oncology Group (ECOG PS), smoking, diabetes, chronic obstructive pulmonary disease (COPD), angina pectoris within 30 days before surgery, congestive heart failure within 30 days before surgery, percutaneous coronary intervention, cerebrovascular disease, risk factors for hemorrhage (anticoagulant therapy, etc.), frailty (5‐item modified frailty index, range; 0–5) [20], TNM stage, intravesical BCG therapy, neoadjuvant and adjuvant chemotherapy, operative time, intraoperative bleeding, type of pelvic lymph node dissection (PLND), type of urinary diversions, type of urinary diversion method (intracorporeal urinary diversion [ICUD] or extracorporeal urinary diversion; [ECUD]), pathological stage, and number of LNs removed. PLND was categorized into the following: standard dissection (obturator and bifurcation of the internal and external iliac artery); extended dissection (the standard + bifurcation of the common iliac artery); super extended dissection (extended + bifurcation of the inferior mesenteric artery); and limited dissection (less than the standard range). Complications were evaluated using the Clavien–Dindo classification and included postoperative delirium, urinary tract infection, superficial and deep incisional surgical site infection, renal dysfunction, postoperative sepsis, anastomotic leakage in the urinary tract, pneumonia, deep vein thrombosis, anastomotic leakage in the gastrointestinal (GI) tract, pulmonary embolism, lower limb compartment syndrome, cardiac arrest requiring resuscitation, central nervous system disorder, myocardial infarction, death within 30 days after surgery, and operative mortality (any death within 90 days) following the National Surgical Quality Improvement Program database from the American College of Surgeons [21]. This study did not include data on recurrence or prognosis; these will be reported in future analyses.

### 2.3 Outcomes

We compared postoperative complications within 30 days between the surgical procedures (RARC vs. ORC and RARC vs. LRC) as well as postoperative complications for each urinary tract diversion (ileal neobladder, ileal conduit, and ureterocutaneostomy) between surgical procedures (RARC vs. ORC, RARC vs. LRC, and RARC‐ICUD vs. RARC‐ECUD). ECUD and ICUD were limited to RARC cases. Multivariable logistic regression analysis was conducted for grade ≥ 3 postoperative complications.

### 2.4 Statistical Analyses

BellCurve for Excel 4.07 (Social Survey Research Information Co. Ltd., Tokyo, Japan), STATA 17 (STATA Corp., TX, USA), and GraphPad Prism 7.00 (GraphPad Software, San Diego, CA, USA) were used for all statistical analyses. Data for quantitative variables were calculated as medians with interquartile ranges (IQRs), and the Mann–Whitney *U* test was conducted to assess differences. Data for categorical variables were calculated as frequencies and percentages, and the chi‐square or Fisher's exact test was used. Logistic regression analysis was conducted to evaluate the effects of grade ≥ 3 postoperative complications. Odds ratios (OR) and 95% confidence intervals (CI) were calculated controlling for potential confounders, including age, sex, ECOG PS, 5‐item modified frailty index, smoking, clinical stage, BCG therapy, neoadjuvant therapy, type of RC and urinary diversion, and extended or more PLND. *p* < 0.05 were considered statistically significant.

## 3 Results

### 3.1 Patient Characteristics

**FIGURE 1:**
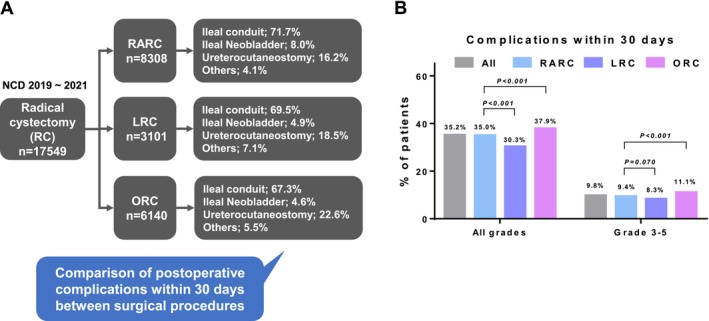
Selection of surgical procedures and comparison of postoperative complications within 30 days between the surgical procedures. A. Selection of surgical procedure and urinary diversion. B. Comparison of postoperative complications within 30 days between the surgical procedures.

The median age of the patients was 73 (IQR: 67–78) years. Overall, 8308, 3101, and 6140 patients underwent RARC, LRC, and ORC, respectively (Table 1, Figure 1A). The number of patients without frailty (ECOG PS 0–1 and 5‐item modified frailty index < 3) was significantly higher in the RARC group than in the LRC and ORC groups (*p* < 0.001); there was no significant difference between the LRC and ORC groups. Significantly more patients in the RARC group received NAC than in the LRC and ORC groups (*p* < 0.001). Additionally, the operative time was longer, blood loss was less, the rate of extended PLND was higher, and lymph node yield was higher in the RARC group compared with the LRC and ORC groups.

**TABLE 1:** Background of patients.

		Total	RARC	LRC	ORC	*p*	RARC vs. ORC	RARC vs. LRC
Number of patients, *n*		17,549	8308	3101	6140			
Age	Years (IQR)	73 (67–78)	73 (67–78)	73 (68–78)	73 (67–78)			
Sex, *n*	Female	3967 (22.6%)	1778 (21.4%)	733 (23.6%)	1456 (23.7%)		< 0.001	0.010
	Male	13,582 (77.4%)	6530 (78.6%)	2368 (76.4%)	4684 (76.3%)			
ECOG PS, *n*	0	12,690 (72.3%)	6182 (74.4%)	2189 (70.6%)	4319 (70.3%)	≤ 1 vs. > 1	0.000	0.002
	1	3248 (18.5%)	1460 (17.6%)	586 (18.9%)	1202 (19.6%)			
	2	860 (4.9%)	355 (4.3%)	172 (5.5%)	333 (5.4%)			
	3	311 (1.8%)	138 (1.7%)	52 (1.7%)	121 (2.0%)			
	4	50 (0.3%)	19 (0.2%)	14 (0.5%)	17 (0.3%)			
	Unknown	390 (2.2%)	154 (1.9%)	88 (2.8%)	148 (2.4%)			
History of smoking, *n*	Positive	9941 (56.6%)	4984 (60.0%)	1616 (52.1%)	3341 (54.4%)		< 0.001	< 0.001
Diabetes, *n*	Positive	3840 (21.9%)	1845 (22.2%)	655 (21.1%)	1340 (21.8%)			
Chronic obstructive pulmonary disease (COPD), *n*	Positive	858 (4.9%)	463 (5.6%)	127 (4.1%)	268 (4.4%)			
Congestive heart failure (within 30 days before surgery), *n*	Positive	64 (0.4%)	25 (0.3%)	7 (0.2%)	32 (0.5%)			
Angina pectoris (within 30 days before surgery), *n*	Positive	206 (1.2%)	92 (1.1%)	35 (1.1%)	79 (1.3%)			
History of percutaneous coronary intervention (PCI), *n*	Positive	590 (3.4%)	306 (3.7%)	95 (3.1%)	189 (3.1%)			
History of cerebrovascular disease, *n*	Positive	876 (5.0%)	411 (4.9%)	155 (5.0%)	310 (5.0%)			
Risk factor for hemorrhage (anticoagulant therapy etc.), *n*	Positive	771 (4.4%)	377 (4.5%)	129 (4.2%)	265 (4.3%)			
Frailty	5‐Item modified frailty index (range: 0–5, IQR)	1 (0–1)	1 (0–1)	1 (0–1)	1 (0–1)			
	5‐Item modified frailty index ≥ 3, *n*	436 (2.5%)	205 (2.5%)	70 (2.3%)	161 (2.6%)		0.559	0.515
cT stage, *n*	cT0	89 (0.5%)	41 (0.5%)	15 (0.5%)	33 (0.5%)	cT ≤ 2 vs. cT ≥ 3	< 0.001	0.555
	cT1	2489 (14.2%)	1216 (14.6%)	479 (15.4%)	794 (12.9%)			
	cT2	7879 (44.9%)	3830 (46.1%)	1354 (43.7%)	2695 (43.9%)			
	cT3	3909 (22.3%)	1821 (21.9%)	653 (21.1%)	1435 (23.4%)			
	cT4	1233 (7.0%)	523 (6.3%)	223 (7.2%)	487 (7.9%)			
	cTX	392 (2.2%)	152 (1.8%)	71 (2.3%)	169 (2.8%)			
	cTa	366 (2.1%)	175 (2.1%)	66 (2.1%)	125 (2.0%)			
	cTis	1192 (6.8%)	550 (6.6%)	240 (7.7%)	402 (6.5%)			
cN stage, *n*	cN0	15,403 (87.8%)	7383 (88.9%)	2756 (88.9%)	5264 (85.7%)	cN0 vs. cN+	< 0.001	0.555
	cN1	1204 (6.9%)	544 (6.5%)	201 (6.5%)	459 (7.5%)			
	cN2	450 (2.6%)	193 (2.3%)	70 (2.3%)	187 (3.0%)			
	cN3	147 (0.8%)	65 (0.8%)	23 (0.7%)	59 (1.0%)			
	cNX	345 (2.0%)	123 (1.5%)	51 (1.6%)	171 (2.8%)			
cM stage, *n*	cM0	16,862 (96.1%)	8040 (96.8%)	2978 (96.0%)	5844 (95.2%)			
	cM1	346 (2.0%)	135 (1.6%)	62 (2.0%)	149 (2.4%)			
	cMX	341 (1.9%)	133 (1.6%)	61 (2.0%)	147 (2.4%)			
History of intravesical BCG therapy, *n*	Positive	3422 (19.5%)	1662 (20.0%)	623 (20.1%)	1137 (18.5%)		0.026	0.964
	Unknown	148 (0.8%)	73 (0.9%)	20 (0.6%)	55 (0.9%)			
Neoadjuvant chemotherapy, *n*	Administered	9447 (53.8%)	4865 (58.6%)	1609 (51.9%)	2973 (48.4%)		< 0.001	< 0.001
	Unknown	32 (0.2%)	14 (0.2%)	10 (0.3%)	8 (0.1%)			
Operative time	Min (IQR)	459 (380–545)	478 (401.5–560)	465 (385–549)	429 (348–517)			
Blood loss	Gram (IQR)	497 (200–1110)	260 (120–500)	350 (171–650)	1300 (800–2063.5)			
Lymph node dissection, *n*	Performed	14,914 (85.0%)	7307 (88.0%)	2539 (81.9%)	5068 (82.5%)		< 0.001	< 0.001
Type of lymph node dissections, *n*	Standard dissection (obturator and bifurcation of the internal and external iliac artery)	8788 (50.1%)	3653 (44.0%)	1573 (50.7%)	3562 (58.0%)	≤ Standard vs. ≥ extended	< 0.001	< 0.001
	Extended dissection (the standard + bifurcation of the common iliac artery)	4771 (27.2%)	3182 (38.3%)	690 (22.3%)	899 (14.6%)			
	Limited dissection (less than the standard range)	1128 (6.4%)	359 (4.3%)	249 (8.0%)	520 (8.5%)			
	Super extended dissection (extended + bifurcation of the inferior mesenteric artery)	127 (0.7%)	76 (0.9%)	9 (0.3%)	42 (0.7%)			
	Others	101 (0.6%)	37 (0.4%)	19 (0.6%)	45 (0.7%)			
	Missing	2634 (15.0%)	1001 (12.0%)	561 (18.1%)	1072 (17.5%)			
Type of urinary diversion methods, *n*	Extracorporeal Urinary Diversion (ECUD)		4156 (50.0%)					
	Intracorporeal Urinary Diversion (ICUD)		3869 (46.6%)					
	None		283 (3.4%)					
Type of urinary diversions, *n*	Ileal conduit	12,245 (69.8%)	5958 (71.7%)	2154 (69.5%)	4133 (67.3%)			
	Ureterocutaneostomy	3311 (18.9%)	1350 (16.2%)	573 (18.5%)	1388 (22.6%)			
	Ileal neobladder	1104 (6.3%)	667 (8.0%)	153 (4.9%)	284 (4.6%)			
	Colonic conduit	26 (0.1%)	10 (0.1%)	5 (0.2%)	11 (0.2%)			
	Intermittent self‐catheterization urinary diversion	27 (0.2%)	11 (0.1%)	6 (0.2%)	10 (0.2%)			
	Others	59 (0.3%)	29 (0.3%)	9 (0.3%)	21 (0.3%)			
pT stage, *n*	pT0	771 (4.4%)	396 (4.8%)	149 (4.8%)	226 (3.7%)	pT ≤ 2 vs. pT ≥ 3	< 0.001	0.273
	pT1	1084 (6.2%)	450 (5.4%)	210 (6.8%)	424 (6.9%)			
	pT2a	1003 (5.7%)	425 (5.1%)	177 (5.7%)	401 (6.5%)			
	pT2b	583 (3.3%)	217 (2.6%)	104 (3.4%)	262 (4.3%)			
	pT3a	1255 (7.2%)	471 (5.7%)	246 (7.9%)	538 (8.8%)			
	pT3b	667 (3.8%)	263 (3.2%)	94 (3.0%)	310 (5.0%)			
	pT4a	816 (4.6%)	341 (4.1%)	159 (5.1%)	316 (5.1%)			
	pT4b	97 (0.6%)	17 (0.2%)	9 (0.3%)	71 (1.2%)			
	pTX	277 (1.6%)	124 (1.5%)	45 (1.5%)	108 (1.8%)			
	pTa	357 (2.0%)	162 (1.9%)	74 (2.4%)	121 (2.0%)			
	pTis	1160 (6.6%)	563 (6.8%)	215 (6.9%)	382 (6.2%)			
	ypT0	1875 (10.7%)	1047 (12.6%)	309 (10.0%)	519 (8.5%)	ypT ≤ 2 vs. ypT ≥ 3	0.187	0.016
	ypT1	907 (5.2%)	441 (5.3%)	184 (5.9%)	282 (4.6%)			
	ypT2a	909 (5.2%)	450 (5.4%)	134 (4.3%)	325 (5.3%)			
	ypT2b	766 (4.4%)	356 (4.3%)	134 (4.3%)	276 (4.5%)			
	ypT3a	1607 (9.2%)	793 (9.5%)	288 (9.3%)	526 (8.6%)			
	ypT3b	825 (4.7%)	421 (5.1%)	162 (5.2%)	242 (3.9%)			
	ypT4a	809 (4.6%)	405 (4.9%)	152 (4.9%)	252 (4.1%)			
	ypT4b	67 (0.4%)	24 (0.3%)	9 (0.3%)	34 (0.6%)			
	ypTX	431 (2.5%)	253 (3.0%)	43 (1.4%)	135 (2.2%)			
	ypTa	268 (1.5%)	141 (1.7%)	51 (1.6%)	76 (1.2%)			
	ypTis	983 (5.6%)	534 (6.4%)	143 (4.6%)	306 (5.0%)			
	Unknown	32 (0.2%)	14 (0.2%)	10 (0.3%)	8 (0.1%)			
pN stage, *n*	pN0	5805 (33.1%)	2526 (30.4%)	1076 (34.7%)	2203 (35.9%)			
	pN1	584 (3.3%)	226 (2.7%)	97 (3.1%)	261 (4.3%)			
	pN2	472 (2.7%)	171 (2.1%)	86 (2.8%)	215 (3.5%)			
	pN3	64 (0.4%)	35 (0.4%)	10 (0.3%)	19 (0.3%)			
	pNX	1145 (6.5%)	471 (5.7%)	213 (6.9%)	461 (7.5%)			
	ypN0	7198 (41.0%)	3724 (44.8%)	1234 (39.8%)	2240 (36.5%)			
	ypN1	836 (4.8%)	401 (4.8%)	168 (5.4%)	267 (4.3%)			
	ypN2	660 (3.8%)	350 (4.2%)	97 (3.1%)	213 (3.5%)			
	ypN3	154 (0.9%)	102 (1.2%)	18 (0.6%)	34 (0.6%)			
	ypNX	599 (3.4%)	288 (3.5%)	92 (3.0%)	219 (3.6%)			
	Unknown	32 (0.2%)	14 (0.2%)	10 (0.3%)	8 (0.1%)			
Number of lymph nodes removed, *n*		15 (9–23)	18 (11–27)	13 (7–20)	13 (7–19)			
Adjuvant therapy, *n*	Administered	2273 (13.0%)	1081 (13.0%)	370 (11.9%)	822 (13.4%)		0.551	0.130
	Unknown	214 (1.2%)	107 (1.3%)	45 (1.5%)	62 (1.0%)			

The number of patients with ileal neobladder was significantly higher in the RARC group (8.0%) than in the LRC (4.9%) and ORC (4.6%) groups (*p* < 0.001). Additionally, the number of ileal conduits was significantly higher in the RARC group (71.7%) than in the LRC (69.5%) and ORC (67.3%) groups (*p* < 0.001). Conversely, the number of ureterocutaneostomies was significantly lower in the RARC group (16.2%) than in the LRC (18.5%) and ORC (22.6%) groups (*p* < 0.001).

### 3.2 Comparison of Postoperative Complications Within 30 Days Between the Surgical Procedures

The rate of any‐grade postoperative complications within 30 days was significantly lower in the RARC (35.9%) and LRC (30.3%) groups than in the ORC group (37.9%, *p* < 0.001); it was also significantly lower in the LRC group (30.3%) than in the RARC group (35.9%, *p* < 0.001). Regarding grade ≥ 3 postoperative complications, its incidence was significantly lower in the RARC group (9.4%) than in the ORC group (11.1%, *p* < 0.001), while it was not significantly different between the RARC and LRC groups (8.3%, *p* = 0.070) (Figure 1B).

### 3.3 Comparison of Postoperative Complications Within 30 Days Between Urinary Diversions

**FIGURE 2:**
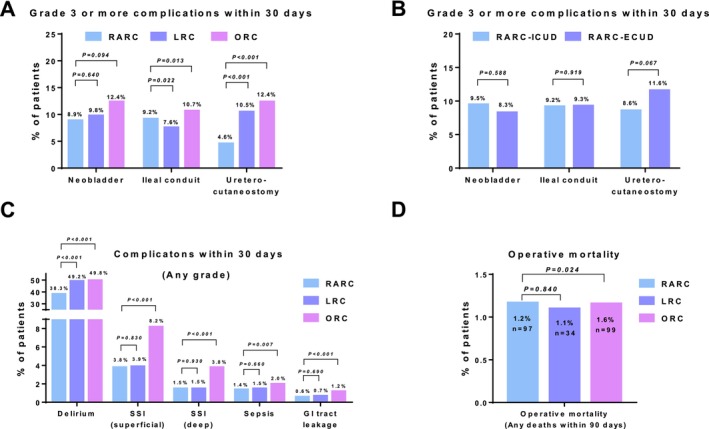
Comparison of postoperative complications and operative mortality. A. Comparison of grade ≥ 3 postoperative complications within 30 days in different urinary diversions between the surgical procedures. B. Comparison of grade ≥ 3 postoperative complications within 30 days in different urinary diversions between the RARC‐ICUD and RARC‐ECUD groups. C. Comparison of any grade of postoperative complications within 30 days between the surgical procedures. D. Comparison of operative mortality (any death within 90 days) between the surgical procedures.

Table 2 presents the postoperative complications within 30 days. The incidence of grade ≥ 3 complications in patients with an ileal conduit was significantly lower in the RARC (9.2%) and LRC (7.6%) groups than in the ORC group (10.7%). RARC with ureterocutaneostomy resulted in a significantly lower rate of grade ≥ 3 complications (4.6%) than LRC (10.5%) and ORC (12.4%). However, the incidence of grade ≥ 3 complications in patients with an ileal neobladder was not significantly different between the RARC (8.9%), LRC (9.8%), and ORC (12.4%) groups (Figure 2A). There was no significant difference between the surgical procedures in terms of the urinary diversion methods (ICUD vs. ECUD) (Figure 2B).

**TABLE 2:** Postoperative complications within 30 days.

Complications within 30 days		Total	RARC	LRC	ORC	RARC vs. ORC	RARC vs. LRC
Number of patients, *n*		17,549	8308	3101	6140		
Clavien–Dindo classification, *n*	No complications	11,319 (64.5%)	5379 (64.7%)	2151 (69.4%)	3789 (61.7%)	< 0.001	< 0.001
	Grade 1–2	4455 (25.4%)	2127 (25.6%)	683 (22.0%)	1645 (26.8%)		
	Grade 3–5	1720 (9.8%)	783 (9.4%)	258 (8.3%)	679 (11.1%)		
	Missing	55 (0.3%)	19 (0.2%)	9 (0.3%)	27 (0.4%)		
Postoperative delirium, *n*	Any grade	7765 (44.2%)	3182 (38.3%)	1527 (49.2%)	3056 (49.8%)	< 0.001	< 0.001
Urinary tract infection, *n*	Any grade	2399 (13.7%)	1127 (13.6%)	379 (12.2%)	893 (14.5%)	0.094	0.062
Surgical site infection (superficial incisional), *n*	Any grade	939 (5.4%)	317 (3.8%)	121 (3.9%)	501 (8.2%)	< 0.001	0.83
Renal disfunction, *n*	Any grade	714 (4.1%)	304 (3.7%)	136 (4.4%)	274 (4.5%)	0.016	0.080
Surgical site infection (deep incisional), *n*	Any grade	408 (2.3%)	126 (1.5%)	48 (1.5%)	234 (3.8%)	< 0.001	0.93
Postoperative sepsis, *n*	Any grade	293 (1.7%)	120 (1.4%)	48 (1.5%)	125 (2.0%)	0.007	0.66
Anastomotic leakage in urinary tract, *n*	Any grade	221 (1.3%)	111 (1.3%)	26 (0.8%)	84 (1.4%)	0.88	0.033
Pneumonia, *n*	Any grade	216 (1.2%)	114 (1.4%)	38 (1.2%)	64 (1.0%)	0.079	0.58
Deep vein thrombosis, *n*	Any grade	177 (1.0%)	85 (1.0%)	24 (0.8%)	68 (1.1%)	0.62	0.24
Anastomotic leakage in gastrointestinal tract, *n*	Any grade	145 (0.8%)	50 (0.6%)	21 (0.7%)	74 (1.2%)	< 0.001	0.69
Pulmonary embolism, *n*	Any grade	79 (0.5%)	42 (0.5%)	7 (0.2%)	30 (0.5%)	0.91	0.052
Lower‐limb compartment syndrome, *n*	Any grade	74 (0.4%)	37 (0.4%)	17 (0.5%)	20 (0.3%)	0.28	0.45
Cardiac arrest requiring resuscitation, *n*	Any grade	71 (0.4%)	37 (0.4%)	10 (0.3%)	24 (0.4%)	0.70	0.41
Central nervous system disorder, *n*	Any grade	51 (0.3%)	21 (0.3%)	7 (0.2%)	23 (0.4%)	0.22	1.00
Myocardial infarction, *n*	Any grade	15 (0.1%)	7 (0.1%)	2 (0.1%)	6 (0.1%)	0.79	1.00
Death within 30 days after surgery, *n*		78 (0.4%)	35 (0.4%)	10 (0.3%)	33 (0.5%)	0.33	0.51
Operative mortality (within 90 days), *n*		230 (1.3%)	97 (1.2%)	34 (1.1%)	99 (1.6%)	0.024	0.84

### 3.4 Detailed Comparison of Postoperative Complications Between the Surgical Procedures

We compared the incidence of any‐grade postoperative complications in each category between RARC versus ORC and RARC versus LRC. RARC resulted in significantly lower rates of delirium, surgical site infection, sepsis, and GI tract leakage than ORC. Additionally, RARC resulted in lower rates of delirium than LRC (Figure 2C). The operative mortality (any death within 90 days) was also significantly lower in RARC (1.2%) than ORC (1.6%, *p* = 0.024), but there was no significant difference between RARC and LRC (1.1%, *p* = 0.840) (Figure 2D).

### 3.5 Multivariable Logistic Regression Analysis for Grade ≥ 3 Postoperative Complications

Multivariable logistic regression analysis revealed that the incidence of grade ≥ 3 postoperative complications was significantly lower in the RARC (OR 0.83, 95% CI 0.72–0.95, *p* = 0.005) and LRC (OR 0.72, 95% CI 0.61–0.85, *p* < 0.001) groups. Meanwhile, age, sex, ECOG PS > 1, 5‐item modified frailty index, and history of smoking had a significantly higher rate of grade ≥ 3 postoperative complications (Table 3).

**TABLE 3:** Multivariable logistic regression analysis.

		OR	*p*	95% CI
Age	≥ 75 years	1.18	0.003	1.06–1.32
Sex	Male	1.43	< 0.001	1.23–1.66
ECOG PS	> 1	1.24	0.029	1.02–1.51
5‐Item modified frailty index	≥ 3	1.37	0.042	1.01–1.85
History of smoking	Yes	1.15	0.020	1.02–1.29
cT	3 or 4	1.00	0.958	0.89–1.13
cN	Positive	0.95	0.609	0.79–1.15
cM	Positive	0.79	0.191	0.55–1.13
History of BCG therapy	Yes	1.07	0.354	0.93–1.22
Neoadjuvant chemotherapy	Underwent	1.02	0.738	0.91–1.14
Type of RC	ORC	Reference (1.00)		
	RARC	0.83	0.005	0.72–0.95
	LRC	0.72	< 0.001	0.61–0.85
Extended PLND	Underwent	1.01	0.909	0.89–1.14
Type of urinary diversion	Ureterocutaneostomy	1.00	1.000	1.00–1.00
	Ileal conduit	0.89	0.095	0.78–1.02
	Neobladder	0.95	0.681	0.75–1.21
	Other urinary diversions	0.91	0.784	0.45–1.82

## 4 Discussion

We investigated the safety of RARC compared with LRC or ORC using the Japanese National Clinical Database, which included 17,549 cases of MIBC. As the Japanese NCD includes nationwide big data that covers 90%–95% of RC performed in Japan, it is one of the largest real‐world big data worldwide [18, 19]. Although there was significant selection bias between the surgical procedures, RARC and LRC resulted in significantly lower rates of any‐grade and grade ≥ 3 postoperative complications than ORC. This suggests that the real‐world advantage of RARC and LRC over ORC is the lower rate of postoperative complications. On the contrary, the incidences of urinary tract infections (RARC 13.6% vs. LRC 12.2%, *p* = 0.062) and pulmonary embolisms (RARC 0.5% vs. LRC 0.2%, *p* = 0.052) showed marginal differences. Although the UTI difference reached borderline significance, the absolute difference of 1.4% is probably clinically meaningful, possibly reflecting the large sample size. Additionally, the number of pulmonary embolism events was extremely low, limiting any clinical interpretation. Prognostic data are currently being collected and were not available for inclusion in this study. Analysis of long‐term follow‐up outcomes is ongoing, and evaluation of overall survival and recurrence‐free survival between the different surgical approaches will commence shortly.

Selection bias for surgical modality cannot be ignored in real‐world practice. Our findings revealed a significant difference in PS, frailty, NAC use, and selection of urinary diversions between the groups. This indicates that high‐intensity treatment is more common in the RARC group based on NAC use and the frequency of ileal neobladder creation. Higher rates of extended PLND and higher LN yields in the RARC group compared with the LRC and ORC groups may also indicate the advantage of surgical manipulation. Although there were differences in patient characteristics between the groups, RARC was still performed in patients with COPD and cardiovascular disease who might not be suitable for RARC. As the number of older patients undergoing RARC has increased due to its less invasive nature [22, 23], its indications are also expanding to include the elderly and patients with several complications. The most substantial source of selection bias stems from inter‐institutional differences—specifically, whether a facility has access to robot‐assisted surgical systems. While many high‐volume centers have adopted these systems, some mid‐sized community hospitals have not. Even in institutions equipped with robotic systems, the choice of surgical approach often depends on the type of abdominal procedure and the patient's history of complications. Nevertheless, the advantages of RARC, including reduced blood loss and lower rates of wound infection, are well established, and many institutions appear to be actively adopting this technique. Although selection bias exists, the widespread use of robot‐assisted surgical equipment has resulted in a wider acceptance of RARC as a stable procedure during the perioperative period. As RARC is not yet established as the gold standard and is currently limited to a minority of high‐volume centers—mainly due to cost constraints—future longitudinal analyses are warranted to clarify how selection bias may evolve in real‐world practice.

Several studies explored the differences between RARC and ORC, with many reporting similar complication rates and oncological outcomes except for selected perioperative complications [3, 12, 24, 25, 26, 27, 28, 29]. A systematic review and meta‐analysis using randomized controlled trials revealed that RARC was associated with lesser blood loss, shorter hospital stays, and fewer thromboembolic events [12, 30]. In our study, multivariate logistic regression analysis revealed that RARC and LRC resulted in a significant reduction in grade ≥ 3 postoperative complications after adjusting for age, sex, poor ECOG PS, frailty, and smoking history, similar to a previous report. These results suggest that the main advantages of RARC may be limited to its high operability and low rate of postoperative complications [12, 26]. In this study, the incidence of delirium, surgical site infection, sepsis, and intestinal leakage was significantly lower following RARC and LRC than ORC, and fewer grade ≥ 3 postoperative complications occurred regardless of the type of urinary diversion. Additionally, RARC resulted in significantly fewer cases of delirium than LRC and ORC, suggesting that the surgical invasiveness of ORC may result in postoperative complications. However, comparisons of individual postoperative complications in this study should be interpreted with caution due to the selection bias for the surgical procedures and facilities. Accordingly, the potential advantages of RARC over ORC might be limited to selected perioperative complications, but it may vary depending on the facilities and regions.

Some complications occurred less with LRC than with RARC, including anastomotic leakage in the GI tract (1.3% vs. 0.8%, respectively, *p* = 0.33), urinary tract infection (13.6% vs. 12.2%, respectively, *p* = 0.062), pulmonary embolism (0.5% vs. 0.2%, respectively, *p* = 0.052), and operative mortality (1.2% vs. 1.1%, respectively, *p* = 0.052). The incidence of postoperative complications following LRC is comparable to that of RARC because LRC is performed by a limited number of experts and at facilities that do not have robot‐assisted surgical equipment.

There was no significant difference in the incidence of grade ≥ 3 postoperative complications between ICUD and ECUD, indicating that the advantage of ICUD may be limited. ICUD (especially ileal neobladder) reportedly requires complicated techniques and a longer operative time, which may be related to its limited advantages [26, 29]. Meanwhile, ICUD is reportedly better than ECUD in terms of specific postoperative complications [24, 25, 27, 31]. Nevertheless, the benefits of ICUD remain controversial. Future studies need to investigate the advantages of postoperative complications associated with RARC and ICUD in controlling selection biases in real‐world practice.

**FIGURE 3:**
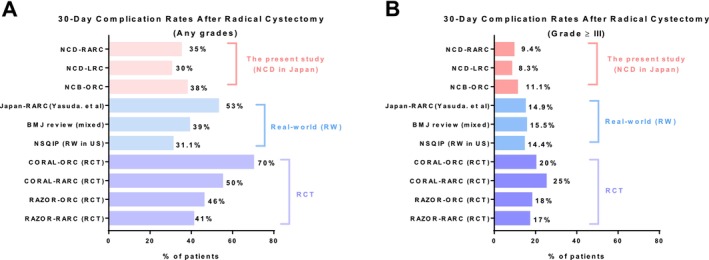
30‐Day postoperative complication rates reported in clinical trials and real‐world studies. A. 30‐Day any‐grade postoperative complication rates after radical cystectomy. B. 30‐Day grade ≥ 3 postoperative complication rates after radical cystectomy.

The frequency of postoperative complications reported in NCD registry‐based studies in Japan warrants further discussion. The definition of operative mortality is “all 30‐day deaths + in‐hospital deaths” (the Japanese NCD registry design guarantees a minimum follow‐up period of 90 days). However, postoperative complications following RARC are recorded for up to 90 days, whereas data for ORC and LRC are limited to a 30‐day follow‐up. Figure 3 summarizes 30‐day postoperative complications following radical cystectomy reported in clinical trials and real‐world studies. The reported rates are inherently lower than those in studies assessing complications over a 90‐day period. In randomized controlled trials (RCTs), 30‐day postoperative complications were reported in 41%–70% of cases (any grade) and 17%–25% for grade ≥ 3 [11, 32]. In real‐world studies, complication rates ranged from 31.1% to 53% (any grade) and 14.4% to 15.5% (grade ≥ 3) [10, 33, 34, 35]. In comparison, the rates observed in our study—30%–38% for any‐grade complications and 9.4%–11.1% for severe complications—appear to fall within or slightly below the range reported in other real‐world studies. It is also important to note that surgical complications in clinical practice are not always systematically captured and may thus be underreported compared to RCTs. Furthermore, existing classification systems for surgical complications may underrepresent low‐grade events, contributing to an overall lower reported complication rate [36]. Another potential factor is that the NCD registry includes only in‐hospital complications, which may further underestimate true incidence rates [37]. Additionally, since radical cystectomies are predominantly performed at high‐volume centers, the possibility of facility‐related bias must also be considered [37]. For these reasons, the frequency of postoperative complications may be underestimated in NCD registry‐based studies in Japan.

The cost of RARC varies substantially depending on the country, insurance system, and surgical volume [38, 39, 40, 41, 42]. In Japan, analysis of the Diagnosis Procedure Combination (DPC) database showed no statistically significant cost difference between RARC and LRC when excluding disposable instruments [43]. Moreover, reports suggest that reduced complications and readmission rates associated with RARC may offset the initial resource use within two years [44]. Overall, the actual cost‐effectiveness of RARC depends on how capital costs are amortized and the extent to which improved postoperative outcomes reduce downstream healthcare utilization. In high‐volume centers, the cost difference tends to diminish or may even be reversed—an important consideration for evaluating surgical strategy.

The COVID‐19 pandemic between 2020 and 2023 significantly impacted Japanese healthcare. This study was conducted during the COVID‐19 pandemic (2019–2022), but the number of RC cases did not decrease (4146, 4665, and 4677 cases in 2019, 2020, and 2021, respectively) [18]. Conversely, the number of RARC cases increased (1416, 2021, and 2343 cases in 2019, 2020, and 2021, respectively) [18]. As MIBC has a poor prognosis, surgery is necessary and cannot be delayed. Furthermore, despite the COVID‐19 pandemic, the minimally invasive procedures of RARC have been widely adopted and evaluated. Moreover, robotic surgery does not spread COVID‐19. RARC is expected to become the standard treatment for MIBC.

This study has several limitations. Although our study had a large number of patients, it was difficult to conduct additional analyses as we did not have access to raw data other than the planned endpoints. Additionally, it was not possible to analyze the prognosis pending the accumulation of future data. Furthermore, interinstitutional bias must be considered because RARC is performed by high‐volume centers and experienced doctors; we did not have access to that information. Regarding LRC, there is a strong selection bias because it is only performed at facilities that do not have robot‐assisted surgical equipment. Another limitation is that small differences can be detected as significant differences when analyzing many cases. As this study did not include data on the implementation of Enhanced Recovery After Surgery (ERAS) protocols, it remains unclear whether ERAS was applied. We were unable to assess complications occurring beyond 90 days, as the Japanese NCD registry records postoperative complications only up to 90 days. Nevertheless, using the NCD enabled us to understand the trends in RARC use, contributing to the current literature and future development of surgical management. The accumulation of prognostic data is expected to further clarify the potential advantages of RARC over ORC, including the incidence of uncommon recurrence patterns such as port‐site recurrences and peritoneal carcinomatosis [10, 45].

## 5 Conclusions

RARC resulted in a lower complication rate compared with ORC in real‐world practice. LRC had comparable outcomes to RARC.

## Author Contributions

**Shingo Hatakeyama:** conceptualization (equal), data curation (equal), investigation (equal), methodology (equal), project administration (equal), visualization (equal), writing – original draft (equal), writing – review and editing (equal). **Hiroyuki Yamamoto:** formal analysis (equal), methodology (equal), resources (equal), software (equal). **Hiroshi Kitamura:** data curation (supporting), supervision (equal), validation (equal). **Toshiyuki Kamoto:** conceptualization (equal), supervision (equal). **Eiji Kikuchi:** conceptualization (equal), data curation (equal), investigation (equal), methodology (equal), project administration (equal), supervision (equal), writing – review and editing (equal).

## Ethics Statement

This retrospective, nationwide study was conducted according to the ethical standards of the Declaration of Helsinki. This study was approved by the Ethics and Conflict of Interest Committee of the National Clinical Database and the Hirosaki University School of Medicine (Authorization No. 2022‐084‐1).

## Consent

According to the provisions of the ethics committee and the ethics guidelines in Japan, written informed consent is not required for retrospective and/or observational studies using materials, such as existing documents, and only public disclosure of study information was needed (opt‐out approach).

## Conflicts of Interest

Shingo Hatakeyama received honoraria from Janssen Pharmaceutical KK, Astellas Pharma Inc., AstraZeneca KK, Ono Pharmaceutical Co. Ltd., Bayer AG, Pfizer Inc., Bristol‐Myers Squibb, Merck BioPharma Co. Ltd., Kaneka Corporation, and Nipro Corporation. Hiroyuki Yamamoto is affiliated with the Department of Healthcare Quality Assessment at the University of Tokyo. The department is a social collaboration department supported by grants from the National Clinical Database, Intuitive Surgical Sarl, Johnson & Johnson KK, and Nipro Co. Hiroshi Kitamura reports consulting or advisory roles for Astellas, AstraZeneca, Eisai, Ferring, Janssen, Kissei, and Takeda; honoraria for Astellas, AstraZeneca, Bristol‐Myers Squibb, Chugai, Eisai, Ferring, Johnson & Johnson/Janssen, Nippon Shinyaku, Merck BioPharma, MSD, Nippon Kayaku, Pfizer, Sanofi, and Takeda; research funding for AstraZeneca, Bristol‐Myers Squibb, MSD, and Takeda. Eiji Kikuchi has participated in consulting or advisory roles for Astellas Pharma, AstraZeneca, Ferring Pharma, Merck BioPharma, Janssen Pharma, Chugai Pharma, and MSD and has participated in speakers bureaus for Astellas Pharma, AstraZeneca, Merck BioPharma, Janssen Pharma, MSD, Bristol‐Myers Squibb, Chugai Pharma, Nippon Kayaku, Eisai, Bayer Pharma, Kyorin Pharma, and Takeda Pharma and has received institutional research funding from MSD, Kyorin Pharma, Nippon Kayaku, Chugai Pharma, Astellas Pharma, AstraZeneca, Bristol‐Myers Squibb, and Merck BioPharma.

## Data Availability

Data is not available due to the nature of this nationwide database study. Participants of this study did not agree for their data to be shared publicly.

## References

[1] J. A. Witjes , H. M. Bruins , R. Cathomas , et al., “European Association of Urology Guidelines on Muscle‐Invasive and Metastatic Bladder Cancer: Summary of the 2020 Guidelines,” European Urology 79, no. 1 (2021): 82–104, 10.1016/j.eururo.2020.03.055. 32360052

[2] H. Matsumoto , K. Shiraishi , H. Azuma , et al., “Clinical Practice Guidelines for Bladder Cancer 2019 Edition by the Japanese Urological Association: Revision Working Position Paper,” International Journal of Urology 27, no. 5 (May 2020): 362–368, 10.1111/iju.14210. 32172529

[3] A. S. Zakaria , F. Santos , A. Dragomir , S. Tanguay , W. Kassouf , and A. G. Aprikian , “Postoperative Mortality and Complications After Radical Cystectomy for Bladder Cancer in Quebec: A Population‐Based Analysis During the Years 2000‐2009,” Canadian Urological Association Journal 8, no. 7–8 (2014): 259–267, 10.5489/cuaj.1997. 25210550 PMC4137011

[4] J. Alfred Witjes , H. Max Bruins , A. Carrion , et al., “European Association of Urology Guidelines on Muscle‐Invasive and Metastatic Bladder Cancer: Summary of the 2023 Guidelines,” European Urology 85, no. 1 (2024): 17–31, 10.1016/j.eururo.2023.08.016. 37858453

[5] H. Matsumoto , K. Shiraishi , H. Azuma , et al., “Clinical Practice Guidelines for Bladder Cancer 2019 Update by the Japanese Urological Association: Summary of the Revision,” International Journal of Urology 27, no. 9 (2020): 702–709, 10.1111/iju.14281. 32564429

[6] B. H. Bochner , G. Dalbagni , K. H. Marzouk , et al., “Randomized Trial Comparing Open Radical Cystectomy and Robot‐Assisted Laparoscopic Radical Cystectomy: Oncologic Outcomes,” European Urology 74, no. 4 (2018): 465–471, 10.1016/j.eururo.2018.04.030. 29784190 PMC6697266

[7] W. Chen , M. Yokoyama , Y. Waseda , et al., “Surgical Outcomes of Robot‐Assisted Radical Cystectomy in Octogenarian or Older Patients: A Japanese Nationwide Study,” International Journal of Urology 30, no. 11 (2023): 1014–1019, 10.1111/iju.15250. 37470427

[8] J. Kamei , K. Endo , M. Yamazaki , et al., “Lower Bleeding Volume Contributes to Decreasing Surgical Site Infection in Radical Cystectomy: A Propensity Score‐Matched Comparison of Open Versus Robot‐Assisted Radical Cystectomy,” International Journal of Urology 31, no. 4 (2024): 430–437, 10.1111/iju.15382. 38173290 PMC11524114

[9] D. Sowanthip , K. Zennami , T. Bejrananda , et al., “Older Versus Younger Patients in Robot‐Assisted Radical Cystectomy With Intracorporeal Ileal Conduit Comparing Safety and Clinical Outcomes,” International Journal of Urology 31, no. 4 (2024): 370–378, 10.1111/iju.15377. 38180102

[10] Y. Yasuda , N. Numao , R. Fujiwara , et al., “Surgical Outcomes and Predictive Value for Major Complications of Robot‐Assisted Radical Cystectomy of Real‐World Data in a Single Institution in Japan,” International Journal of Urology 31, no. 7 (2024): 724–729, 10.1111/iju.15447. 38477173

[11] D. J. Parekh , I. M. Reis , E. P. Castle , et al., “Robot‐Assisted Radical Cystectomy Versus Open Radical Cystectomy in Patients With Bladder Cancer (RAZOR): An Open‐Label, Randomised, Phase 3, Non‐Inferiority Trial,” Lancet 391, no. 10139 (2018): 2525–2536, 10.1016/s0140-6736(18)30996-6. 29976469

[12] P. Khetrapal , J. K. L. Wong , W. P. Tan , et al., “Robot‐Assisted Radical Cystectomy Versus Open Radical Cystectomy: A Systematic Review and Meta‐Analysis of Perioperative, Oncological, and Quality of Life Outcomes Using Randomized Controlled Trials,” European Urology 84, no. 4 (2023): 393–405, 10.1016/j.eururo.2023.04.004. 37169638

[13] K. I. Birkeland , J. Bodegard , J. W. Eriksson , et al., “Heart Failure and Chronic Kidney Disease Manifestation and Mortality Risk Associations in Type 2 Diabetes: A Large Multinational Cohort Study,” Diabetes, Obesity & Metabolism 22, no. 9 (2020): 1607–1618, 10.1111/dom.14074. PMC7496468 32363737

[14] H. Matsunaga , K. Ito , M. Akiyama , et al., “Transethnic Meta‐Analysis of Genome‐Wide Association Studies Identifies Three New Loci and Characterizes Population‐Specific Differences for Coronary Artery Disease,” Circulation: Genomic and Precision Medicine 13, no. 3 (2020): e002670, 10.1161/circgen.119.002670. 32469254

[15] S. Yajima , S. Hata , N. Masumori , et al., “Patient Preferences for Post‐Radical Cystectomy Treatment in Muscle‐Invasive Bladder Cancer: A Discrete Choice Experiment in Japan,” International Journal of Urology 32 (2025): 688–697, 10.1111/iju.70032. 40062520 PMC12146242

[16] E. Zeng , M. Saucke , B. Pati , A. Rose , E. Alagoz , and K. A. Richards , “Improving Rural Access to Urological Healthcare: Patient Experiences With Cystectomy and Urinary Diversion,” International Journal of Urology 32 (2025): 758–760, 10.1111/iju.70033. 40035264 PMC12146243

[17] A. Tomotaki , H. Kumamaru , H. Hashimoto , et al., “Evaluating the Quality of Data From the Japanese National Clinical Database 2011 via a Comparison With Regional Government Report Data and Medical Charts,” Surgery Today 49, no. 1 (2019): 65–71, 10.1007/s00595-018-1700-5. 30088123

[18] E. Kikuchi , H. Yamamoto , T. Yasui , et al., “The First Detailed Annual Record on the National Clinical Database Urology Division in Japan: A Report on Five Surgical Procedures,” International Journal of Urology 31, no. 12 (2024): 1344–1355, 10.1111/iju.15561. 39154336

[19] T. Yasui , E. Kikuchi , H. Yamamoto , et al., “Annual Record on the Number of General Urological Surgeries Registered in the National Clinical Database System Between April 2018 and December 2021 in Japan,” International Journal of Urology 31, no. 11 (2024): 1256–1262, 10.1111/iju.15551. 39105577

[20] N. J. Sathianathen , S. Jarosek , N. Lawrentschuk , D. Bolton , and B. R. Konety , “A Simplified Frailty Index to Predict Outcomes After Radical Cystectomy,” European Urology Focus 5, no. 4 (2019): 658–663, 10.1016/j.euf.2017.12.011. 29366857

[21] A. Tran , L. R. Putnam , J. C. Lipham , and S. Shiraga , “Utility of the mFI‐5 as a Predictor of Post‐Operative Outcomes Following Gastrectomy for Gastric Cancer: An ACS‐NSQIP Analysis,” Surgical Endoscopy 38, no. 10 (2024): 5922–5928, 10.1007/s00464-024-11103-3. 39046494 PMC11458691

[22] H. Iwamoto , T. Yumioka , N. Yamaguchi , et al., “Robot‐Assisted Radical Cystectomy Is a Promising Alternative to Open Surgery in the Japanese Population With a High Rate of Octogenarians,” International Journal of Clinical Oncology 21, no. 4 (2016): 756–763, 10.1007/s10147-016-0950-8. 26792433

[23] A. Yu , Y. Wang , M. Mossanen , et al., “Robotic‐Assisted Radical Cystectomy is Associated With Lower Perioperative Mortality in Octogenarians,” Urologic Oncology: Seminars and Original Investigations 40, no. 4 (2022): 163e19–163e23, 10.1016/j.urolonc.2021.08.027. 34602361

[24] S. An , L. Shi , Y. Liu , L. Ren , K. Zhang , and M. Zhu , “Comparison of Extracorporeal and Intracorporeal Urinary Diversion After Robot‐Assisted Radical Cystectomy for Bladder Cancer: A Meta‐Analysis,” American Journal of Men's Health 18, no. 5 (2024): 15579883241274866, 10.1177/15579883241274866. PMC11526166 39462910

[25] P. E. Gabriel , U. Pinar , L. Lenfant , et al., “Comparative Effectiveness of Robot‐Assisted Radical Cystectomy With Intracorporeal Urinary Diversion vs Open Radical Cystectomy for Bladder Cancer,” BJU International 135, no. 3 (2025): 517–527, 10.1111/bju.16565. 39433445 PMC11842888

[26] G. Novara , J. W. Catto , T. Wilson , et al., “Systematic Review and Cumulative Analysis of Perioperative Outcomes and Complications After Robot‐Assisted Radical Cystectomy,” European Urology 67, no. 3 (2015): 376–401, 10.1016/j.eururo.2014.12.007. 25560798

[27] R. S. Flammia , L. C. Licari , E. Bologna , et al., “Comparative Outcomes of Open Radical Cystectomy vs. Robot‐Assisted Approaches With Intracorporeal and Extracorporeal Urinary Diversion: A Meta‐Analysis and Network Meta‐Analysis of Perioperative and Quality of Life Outcomes,” Journal of Clinical Medicine 13, no. 8 (2024): jcm13082421, 10.3390/jcm13082421. PMC11051502 38673693

[28] V. Venkatramani , I. M. Reis , E. P. Castle , et al., “Predictors of Recurrence, and Progression‐Free and Overall Survival Following Open Versus Robotic Radical Cystectomy: Analysis From the RAZOR Trial With a 3‐Year Followup,” Journal of Urology 203, no. 3 (2020): 522–529, 10.1097/ju.0000000000000565. 31549935 PMC7487279

[29] Z. Dalimov , U. Iqbal , Z. Jing , et al., “Intracorporeal Versus Extracorporeal Neobladder After Robot‐Assisted Radical Cystectomy: Results From the International Robotic Cystectomy Consortium,” Urology 159 (2022): 127–132, 10.1016/j.urology.2021.10.012. 34710397

[30] L. Cella , G. Basile , S. Moretto , et al., “Robotic Assisted vs Open Radical Cystectomy: An Updated Systematic Review and Meta‐Analysis,” Journal of Robotic Surgery 18, no. 1 (2024): 277, 10.1007/s11701-024-02026-1. 38961035

[31] H. Wang , W. Wang , X. Wang , et al., “Intracorporeal Urinary Diversion Offers the Advantage of Delaying Postoperative Renal Function Injury in Patients Undergoing Robot‐Assisted Radical Cystectomy,” Frontiers in Oncology 14 (2024): 1435050, 10.3389/fonc.2024.1435050. 39296976 PMC11408127

[32] M. S. Khan , C. Gan , K. Ahmed , et al., “A Single‐Centre Early Phase Randomised Controlled Three‐Arm Trial of Open, Robotic, and Laparoscopic Radical Cystectomy (CORAL),” European Urology 69, no. 4 (2016): 613–621, 10.1016/j.eururo.2015.07.038. 26272237

[33] S. L. Maibom , U. N. Joensen , A. M. Poulsen , H. Kehlet , K. Brasso , and M. A. Røder , “Short‐Term Morbidity and Mortality Following Radical Cystectomy: A Systematic Review,” BMJ Open 11, no. 4 (2021): e043266, 10.1136/bmjopen-2020-043266. PMC8054090 33853799

[34] G. Gandaglia , B. Varda , A. Sood , et al., “Short‐Term Perioperative Outcomes of Patients Treated With Radical Cystectomy for Bladder Cancer Included in the National Surgical Quality Improvement Program (NSQIP) Database,” Canadian Urological Association Journal 8, no. 9–10 (2014): E681–E687, 10.5489/cuaj.2069. 25408807 PMC4216299

[35] J. M. Knorr , K. J. Ericson , J. H. Zhang , et al., “Comparison of Major Complications at 30 and 90 Days Following Radical Cystectomy,” Urology 148 (2021): 192–197, 10.1016/j.urology.2020.08.038. 32888983

[36] C. Soliman , N. J. Sathianathen , B. C. Thomas , et al., “A Systematic Review of Intra‐ and Postoperative Complication Reporting and Grading in Urological Surgery: Understanding the Pitfalls and a Path Forward,” European Urology Oncology 6, no. 4 (2023): 378–389, 10.1016/j.euo.2023.01.002. 36697322

[37] S. Kunisawa , “Postoperative Mortality Analysis on Nationwide Data From Diagnosis Procedure Combination Database in Japan,” PLoS One 18, no. 6 (2023): e0286264, 10.1371/journal.pone.0286264. 37289744 PMC10249857

[38] Y. Morii , T. Osawa , T. Suzuki , et al., “Cost Comparison Between Open Radical Cystectomy, Laparoscopic Radical Cystectomy, and Robot‐Assisted Radical Cystectomy for Patients With Bladder Cancer: A Systematic Review of Segmental Costs,” BMC Urology 19, no. 1 (2019): 110, 10.1186/s12894-019-0533-x. 31703573 PMC6842244

[39] J. J. Leow , S. W. Reese , W. Jiang , et al., “Propensity‐Matched Comparison of Morbidity and Costs of Open and Robot‐Assisted Radical Cystectomies: A Contemporary Population‐Based Analysis in the United States,” European Urology 66, no. 3 (2014): 569–576, 10.1016/j.eururo.2014.01.029. 24491306

[40] S. Dixon , H. Hill , L. Flight , et al., “Cost‐Effectiveness of Robot‐Assisted Radical Cystectomy vs Open Radical Cystectomy for Patients With Bladder Cancer,” JAMA Network Open 6, no. 6 (2023): e2317255, 10.1001/jamanetworkopen.2023.17255. 37389878 PMC10314306

[41] A. Gastecka , A. Hnatyszyn‐Dzikowska , P. Hejka , et al., “Cost Comparison of Laparoscopic Versus Robot‐Assisted Radical Cystectomy,” Health Policy and Technology 7, no. 4 (2018): 420–426, 10.1016/j.hlpt.2018.10.008.

[42] F. Machleid , J. Ho‐Wrigley , A. Chowdhury , A. Paliah , H. L. Poon , and E. Pizzo , “Cost‐Utility Analysis of Robotic‐Assisted Radical Cystectomy for Bladder Cancer Compared to Open Radical Cystectomy in the United Kingdom,” PLoS One 17, no. 9 (2022): e0270368, 10.1371/journal.pone.0270368. 36174057 PMC9522012

[43] M. Yokoyama , W. Chen , Y. Waseda , et al., “Comparisons of In‐Hospital Fee and Surgical Outcomes Between Robot‐Assisted, Laparoscopic, and Open Radical Cystectomy: A Japanese Nationwide Study,” Japanese Journal of Clinical Oncology 54, no. 7 (2024): 822–826, 10.1093/jjco/hyae039. 38553780

[44] S. Trecarten , C. Schaefer , A. Elshabrawy , et al., “Two‐Year Resource Utilization of Open vs. Robot‐Assisted Radical Cystectomy: Results From Optum's De‐Identified Clinformatics Data Mart Database,” Urologic Oncology 43 (2025): 391.e21–391.e28, 10.1016/j.urolonc.2025.03.002. 40180848

[45] D. P. Nguyen , B. Hussein Al Awamlh , X. Wu , et al., “Recurrence Patterns After Open and Robot‐Assisted Radical Cystectomy for Bladder Cancer,” European Urology 68, no. 3 (2015): 399–405, 10.1016/j.eururo.2015.02.003. 25709026 PMC4727829

